# Treatment Patterns and Survival among Patients with Metastatic Gastroenteropancreatic Neuroendocrine Tumours in Sweden - a Population-based Register-linkage and Medical Chart Review Study

**DOI:** 10.7150/jca.32381

**Published:** 2019-11-17

**Authors:** Eva Lesén, Daniel Granfeldt, Anthony Berthon, Jérôme Dinet, Aude Houchard, Peter Myrenfors, Åse Björstad, Ingela Björholt, Anna-Karin Elf, Viktor Johanson

**Affiliations:** 1 Former employee of PharmaLex, Gothenburg, Sweden.; 2 PharmaLex, Gothenburg, Sweden.; 3 Former employee of Ipsen, Boulogne-Billancourt, France.; 4 Ipsen, Boulogne-Billancourt, France.; 5 Former employee of Ipsen Nordic, Stockholm, Sweden.; 6 Department of Surgery, Sahlgrenska University Hospital, Gothenburg, Sweden.; 7 Former employee at Department of Surgery, Sahlgrenska University Hospital, Gothenburg, Sweden.

**Keywords:** GEP-NET, metastatic, SSA, surgery, treatment patterns, survival.

## Abstract

**Background:** Gastroenteropancreatic neuroendocrine tumours (GEP-NETs) are neoplasms derived from the endocrine system in the gastrointestinal tract and pancreas. Treatment options include surgery; pharmacological treatments like somatostatin analogues (SSA), interferon alpha, molecular targeted therapy and chemotherapy; and peptide receptor radionuclide therapy.

The objective of this study was to describe treatment patterns and survival among patients with metastatic GEP-NET grade 1 or 2 in Sweden.

**Methods:** Data was obtained via linkage of nationwide registers. Patients diagnosed with metastatic GEP-NET grade 1 or 2 in Sweden between 2005 and 2013 were included (n=811; National population). In addition, medical chart review was performed for the subpopulation diagnosed at Sahlgrenska University Hospital, Gothenburg (n=127; Regional population). Treatment patterns, including treatment sequences, and overall survival were assessed.

**Results:** Most patients had small intestinal NET (76%). In the regional population, 72% had grade 1 tumours; 50% had functioning tumours. The two most common first-line treatments were surgery (57%) and SSA (25%). After first-line surgery, 46% received SSA, while 40% had no further treatment. After first-line SSA, 52% received surgery, while 27% had no further treatment. Overall median survival time from date of diagnosis was 7.0 years (95% CI 6.2-not reached). Among patients with distant metastases, pancreatic NET (vs. small intestinal NET) was associated with poorer survival (HR 1.9; 95% CI 1.1-3.3), as were liver metastases (HR 3.2; 95% CI 1.5-7.0).

**Conclusions:** First-line surgery was typically followed by SSA or no further treatment. Among patients with distant metastases, pancreatic NET or liver metastases were associated with a poorer survival.

## Introduction

Gastroenteropancreatic neuroendocrine tumours (GEP-NETs) are rare and include a heterogeneous group of neoplasms derived from the endocrine system in the gastrointestinal (GI) tract and pancreas, representing approximately 2% of all GI tumours 1.

The incidence of GEP-NET has increased over time, possibly as a result of improved detection and diagnostics 2. The overall annual incidence is estimated to 2.5-3.7 per 100,000; highest for GEP-NETs in the small intestine, followed by rectum, colon and pancreas 3.

Surgery is the primary treatment option; curative surgery should be considered even in the case of metastatic disease, when possible. For more progressed disease, debulking and palliative surgery may reduce symptom load as well as facilitate and improve the outcomes from subsequent treatments. If present, hormonal symptoms can usually be ameliorated with somatostatin analogues (SSA); a second line option is interferon alpha (IFN-alpha). Other treatments for GEP-NET include molecular targeted therapy, chemotherapy and peptide receptor radionuclide therapy (PRRT). For liver metastases, surgical resection, hepatic artery embolization (HAE), selective internal radiation therapy (SIRT) and radiofrequency ablation (RFA) or microwave ablation (MWA) can be performed 4-6.

There are several publications describing real-world treatment patterns for GEP-NET 7-23. The majority of these studies are North American (mainly US), while a few are from Europe and other continents. These publications are based on data from cancer registers, medical chart review, medical claims databases and/or healthcare databases, or a combination of these sources, but a very limited number of the studies have national coverage. The numbers of included patients vary from 25 to 2,250; the vast majority of studies include ≤300 patients. Treatment patterns described in these publications vary widely, but generally include the treatment options presented above. None of the publications describe real-world treatment patterns specific for Sweden.

The overall 5-year survival rate in GEP-NET has been reported as approximately 70%, spanning from 38% for pancreatic NETs to 89% for rectal NETs, and approaching 100% for gastric NETs (type I) 3, 7, 9, 10. The prognosis is influenced by age at diagnosis, tumour size, levels of biomarkers (e.g. chromogranin A [CgA]), Ki 67 index, and the presence of metastases and clinical symptoms 24-28. For pancreatic NET, median survival time has been estimated as 11.3 years for patients with localized disease, and 2.0-3.9 years for those with distant metastases. For patients with locally advanced/metastatic GEP-NET, median survival time has been reported as 12.7 years from initiation of first-line therapy. For NET in the small intestine, median survival time has been estimated as 9.3 years for patients with localized disease and 4.7-7.9 years for those with distant metastases 11, 13, 27-29.

To summarise, publications providing valuable information on treatment patterns and survival for GEP-NET are mostly North American and few European studies exist. In light of this, the objective of the current study was to describe treatment patterns and survival among patients diagnosed with metastatic GEP-NET (grade 1 or 2) in Sweden between 1 July 2005 and 31 December 2013.

## Materials and Methods

### Data sources

#### National population

For the population denoted National population (covering all of Sweden), data was obtained from several national registers covering the entire Swedish population. The high-precision linkage was enabled by the unique personal identification numbers. Patients were selected via the Cancer Register, which includes information on all newly diagnosed tumours. The National Patient Register was used for complementing information on metastatic status; this register includes information on all specialized health care contacts. The Swedish Prescribed Drug Register, established in July 2005, was used for data on prescribed medicines purchased in Swedish pharmacies. The registers are held by the National Board of Health and Welfare.

#### Regional population

In addition to data obtained from national registries as described above, a medical chart review was performed for a subgroup of the National population, denoted the Regional population. The Regional population included patients diagnosed at Sahlgrenska University Hospital in Gothenburg and the medical chart review contributed with in-depth clinical data not available in the national registers. The personal identification numbers for these patients were extracted from the national registers and used for identification of the patients' medical charts. The extraction of data from the medical charts was performed by authors VJ and A-KE according to a predefined and pilot tested data extraction form.

### Eligibility criteria

The study population included patients with a first diagnosis of metastatic GEP-NET (grade 1 or 2) established in Sweden between 1 July 2005 and 31 December 2013. The start of the inclusion period was selected due to changes in the diagnostic coding system in the Cancer Register 2004/2005. Further, the Drug Register was established in July 2005. GEP-NET was defined with the following diagnostic criteria (both criteria were mandatory), according to data in the Cancer Register: 1) International Classification of Diseases for Oncology, 3^rd^ edition (ICD-O/3) codes for gastroenteropancreatic tumour sites: C16-C20, C25, and 2) ICD-O/3 morphological codes for neuroendocrine type: 80133, 80413, 81500, 81503, 81510, 81513, 81521, 81523, 81531, 81533, 81553, 81561, 81563, 82403, 82413, 82421, 82423, 82463, 82493, and 86830 (however, the codes 80133, 81553, 81561, 81563, 82413, 82423, 82463, 86830 were not reported for any patient in the dataset). In order to account for potential variations in morphological coding concerning grade, also codes suggestive of grade 3 disease were included. Based on survival analysis as described below, patients deemed to have graded 3 tumours were excluded. Patients with metastatic GEP-NET at the time of diagnosis were selected based on Tumour, Node, Metastases (TNM) codes in the Cancer Register (N1-3 and/or M1) and/or ICD-10 codes in the National Patient Register (specialised health care visit/admission with diagnostic code C77-C79 within 6 months from the GEP-NET diagnosis). In the resulting dataset, the morphological codes registered at each tumour site were reviewed to exclude patients with inconsistent or invalid diagnoses. Furthermore, the survival among patients with morphological codes suggestive of grade 3 tumours was compared to the survival among patients with other morphological codes (via Kaplan-Meier curves and log-rank tests; data not shown). Patients with morphological codes 80413 and 82463 had a markedly poorer overall survival compared to the other patients (p<0.0001 in both analyses), and were thus excluded.

For the subgroup of patients diagnosed at Sahlgrenska University Hospital in Gothenburg, the eligibility criteria were reviewed once more based on the in-depth data available in the medical charts. Patients still fulfilling the criteria constituted the Regional population.

### Definitions and assessments

The clinical characteristics were described at diagnosis (see Table 1 for detailed definitions). The date of diagnosis corresponds to the date for the first examination forming the basis for the clinical diagnosis.

The following treatment options were assessed: surgical interventions (for the primary tumour site and for metastatic sites), locoregional interventions (HAE and RFA), PRRT, external radiotherapy and pharmacological treatment (SSA, IFN-alpha, chemotherapy, and molecular targeted therapy). The start of treatment was defined as the date of the surgical or medical intervention, or as the date of the first purchase of the prescribed drug, on or after the date of GEP-NET diagnosis.

Survival analyses of overall survival (OS) in relation to clinical characteristics at diagnosis were performed for the National population, and the survival time was defined from the date of diagnosis of metastatic GEP-NET to death from any cause. Information on date of death and cause of death as reported in the Cancer Register was transferred from the Cause of Death Register in Sweden. Patients alive at 31 December 2013 were censored at that date. Survival analyses were also performed in relation to treatment status for the National population. To avoid imposing a survival advantage on patients who have survived long enough to have received treatment (i.e. immortal time bias), these analyses were performed separately for patients who only had a first-line treatment during the observation period, and among patients who also had a second-line treatment during the observation period, respectively. In these analyses, the survival time was defined from the start of first-line treatment, or the start of second-line treatment, respectively.

### Statistical analyses

Standard descriptive statistics were used to summarise the data. Percentages were based on the number of non-missing observations.

Based on the Kaplan-Meier curve, the estimated median survival time (with 95% confidence intervals [CI]) was assessed, along with 1, 2 and 5-year survival rates. Univariate Cox proportional hazards regression was performed for the main effect variables (i.e. clinical characteristics or treatment status, respectively), potential confounders and interaction terms. The proportional hazards assumption was checked (graphically by investigating the plot of log(-log(survival function)) vs log(time) for all variables) and considered satisfied. Based on the results from the univariate modelling, the main effect variables, potential confounders and interaction terms associated with risk of death at p<0.10 were entered into a forward stepwise selection model (Cox regression). The number of variables in the multivariate model was not allowed to exceed the number of events, i.e. deaths, divided by 10, to avoid overfitting the model. The results were presented with Hazard Ratios (HR), 95% CI and p-values. P-values <0.05 were considered statistically significant. For interaction terms that were statistically significant, the multivariate model is presented by stratas.

All analyses were performed in SAS® version 9.4 (Cary, NC, USA).

### Ethical considerations

The study was approved by the Regional Ethical Review Board at the University of Gothenburg (Dnr 218-15). For ethical reasons, the number of patients was presented as “<5” when the exact number of patients was 1-4, so that no individual could be identified.

## Results

### Patient characteristics

**Figure 1:**
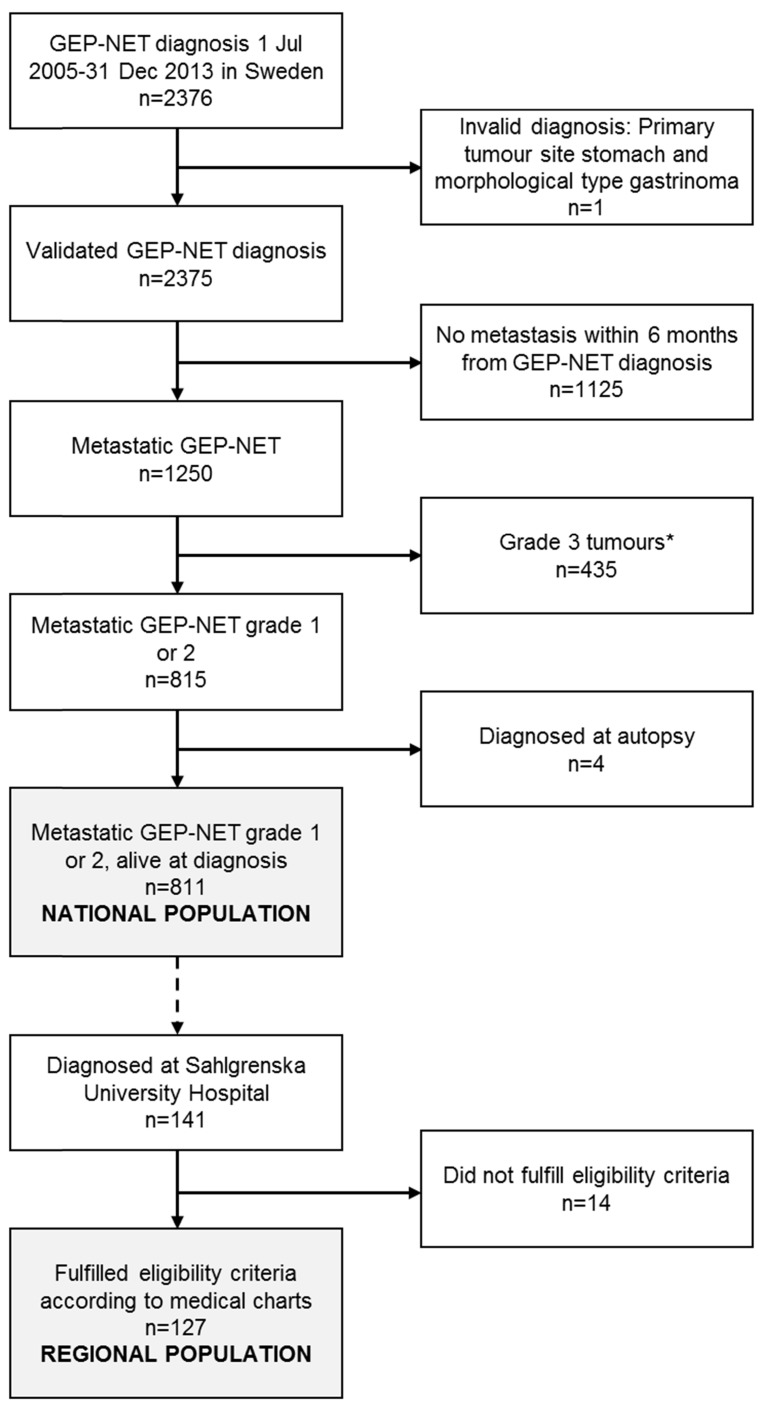
Patient flow-chart, resulting in the National population, including patients diagnosed in all of Sweden, and the Regional population, covering the subgroup of patients diagnosed at Sahlgrenska University Hospital in Gothenburg. GEP-NET: gastroenteropancreatic neuroendocrine tumours. *Patients with grade 3 tumours were excluded based on a comparison of the survival (via Kaplan-Meier curves and log-rank tests) for patients with morphological codes suggestive of grade 3 tumours in relation to the survival among patients with other morphological codes. Morphological codes 80413 and 82463 were excluded (patients with these morphological codes had a markedly poorer overall survival compared to the other patients; p<0.0001 in both analyses).

Figure 1 presents the patient selection flow chart. The National population consisted of 811 patients diagnosed with metastatic GEP-NET (grade 1 or 2) in Sweden 1 July 2005 - 31 December 2013. The extended review of eligibility criteria performed for the subgroup of patients diagnosed at Sahlgrenska University Hospital in Gothenburg (n=141) based on data in the medical charts led to the exclusion of 14 patients: 7 patients had a grade 3 tumour (Ki 67 index >20%), 5 patients had been diagnosed with GEP-NET prior to the inclusion period, 1 patient had been misclassified as having NET, and for 1 patient, NET was merely an incidental finding and was not considered to have given rise to the metastasis or any of the treatments. The Regional population thus consisted of 127 patients.

The clinical characteristics at diagnosis for patients in the National population and in the subgroup of patients in the Regional population, respectively, are presented in Table 1. The mean age at diagnosis in the National population was 66.2 years (SD 12.2 years), and 52.2% were male. The majority of patients had small intestinal NET (76%), followed by pancreatic NET (11%). Among the patients with known metastatic site (n=307), the most common metastatic sites at diagnosis were the liver (60%) and lymph nodes (43%). Carcinoid syndrome had been diagnosed among 36% within 6 months from diagnosis, while 1% had been diagnosed with carcinoid heart disease during the same time span. The median follow-up time was 2.93 years (range 0.01‒8.46 years). In the Regional population, the mean age at diagnosis was 65.6 years (SD 11.7 years), and 50.4% were male. Seventy-four percent of patients had carcinoid syndrome at diagnosis. The liver metastatic burden was considered to be high for 7% of the patients. Half of the patients in the Regional population had a functioning tumour (i.e. experienced hormonal symptoms). The majority of patients had biomarker levels above the upper limit of normal (ULN) for both 5-Hydroxyindoleacetic acid (5-HIAA) and CgA, respectively. A quarter (32 of 116 patients with data available) had used proton-pump inhibitors (PPI) at the time of the CgA measurement. The median follow-up time in the Regional population was 2.52 years (range 0.11‒8.43 years).

### Treatment patterns

#### Overall occurrence of treatments

Table 2 presents the treatments received by patients in the overall National population and in the subgroup of patients in the Regional population, at any time during the observation period. The percentage of patients with any surgery related to metastatic GEP-NET was 72% in the National population and 88% in the Regional population. Surgery of the primary tumour site was more common in small intestinal NET (74%; 95% CI 70-77%) than in pancreatic NET (39%; 95% CI 28-50%); this pattern was also observed in the Regional population (91%; 95% CI 84-96%) vs. (44%; 95% CI 22-69%).

HAE and RFA had been performed among 10% and 9% of patients in the National population, respectively (Table 2). The corresponding figures in the Regional population were 32% (HAE) and 7% (RFA). In the Regional population, HAE was more common among patients with functioning tumours than with non-functioning tumours; 45% (95% CI 33-58%) vs. 18% (95% CI 9-30%), respectively. At least one cycle of PRRT had been performed among 8% of the patients in the National population and 9% in the Regional population.

The most common pharmacological treatment was SSA (Table 2), followed by IFN-alpha, chemotherapy and molecular targeted therapy. SSA was more common among patients with small intestinal NET (National population: 66%; 95% CI 62-69%) than among those with pancreatic NET (39%; 95% CI 28-50%). Contrary, chemotherapy was more common in pancreatic NET (52%; 95% CI 41-63%) than in small intestinal NET (5%; 95% CI 3-7%). These patterns were also observed in the Regional population (data not shown). Patients with functioning tumours had a higher occurrence of SSA use than patients with non-functioning tumours (89% [95% CI 78-95%] vs. 61% [95% CI 47-73%]), and the time to SSA initiation from diagnosis was median 22 (mean 98; 95% CI for mean 27-169) vs. 59 (mean 166; 95% CI for mean 41-291) days, respectively.

#### First- and second-line treatments

The two most common first-line treatments were surgery and SSA, both in the National and in the Regional populations (Table 3). This pattern was observed among patients with small intestinal NET and among those with pancreatic NET (data not shown). Overall, few patients had any of the other treatments as first-line. However, for patients with pancreatic NET in the National population (n=85), 20% had chemotherapy as first-line treatment.

Among the 460 patients in the National population with first-line surgery, the most common second-line treatment was SSA, which was received by 46% of patients and initiated median 1.3 (mean 5.7; 95% CI for mean 4.1-7.3) months after surgery (Table 4). Forty percent received no second-line treatment after surgery during the observation period. In the Regional population, 78% (95% CI 62-89%) of patients with functioning tumours received SSA after first-line surgery; this was 46% (95% CI 30-63%) among those with non-functioning tumours.

In the case of SSA as first-line, surgery was the most common second-line treatment, both in the National population (52%) and in the Regional population (75%) (Table 4).

### Survival

One third (n=265, 33%) of the patients in the National population died during the observation period. Close to half of the deaths (n=127, 48% of all deaths) were considered to be caused by GEP-NET (the ICD-10 code for cause of death was at the same site as that of the primary GEP-NET tumour). The remaining deaths (n=138, 52%) were due to other causes.

The median survival from date of diagnosis (including deaths from any cause) was 7.0 years (95% CI for median 6.2-not reached). The 1-, 2- and 5-year survival rates were 87%, 79% and 63%, respectively.

#### Overall survival and risk of death in relation to clinical characteristics at diagnosis

The median survival by tumour site ranged from 4.2 (95% CI 2.5-not reached) years for other GEP-NET to 7.0 (95% CI 6.5-not reached) years for small intestinal NET. For pancreatic NET, the median survival was 4.3 (95% CI 2.8-not reached) years.

**Figure 2:**
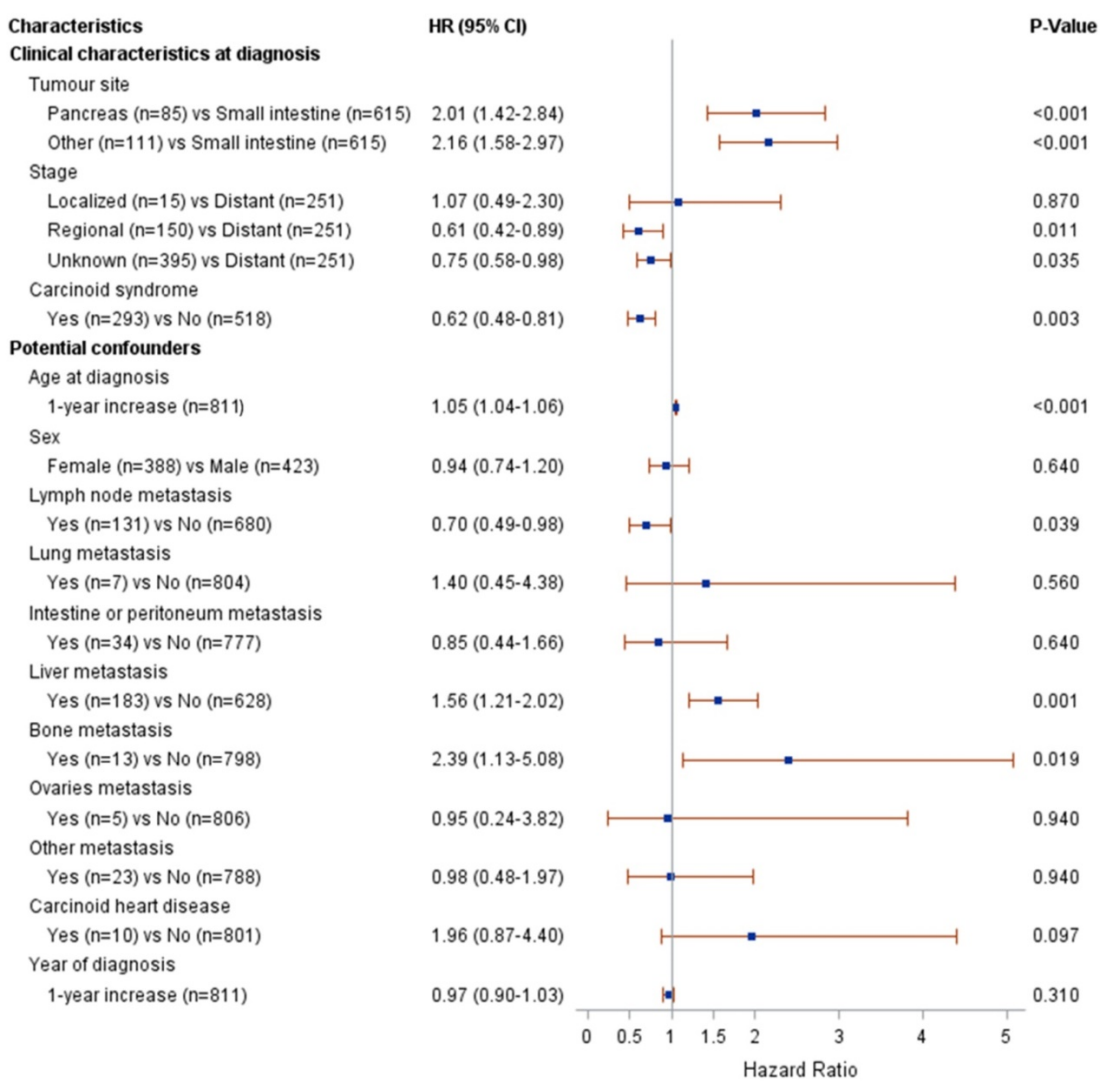
Results from univariate Cox modelling of clinical characteristics and risk of death.

**Figure 3:**
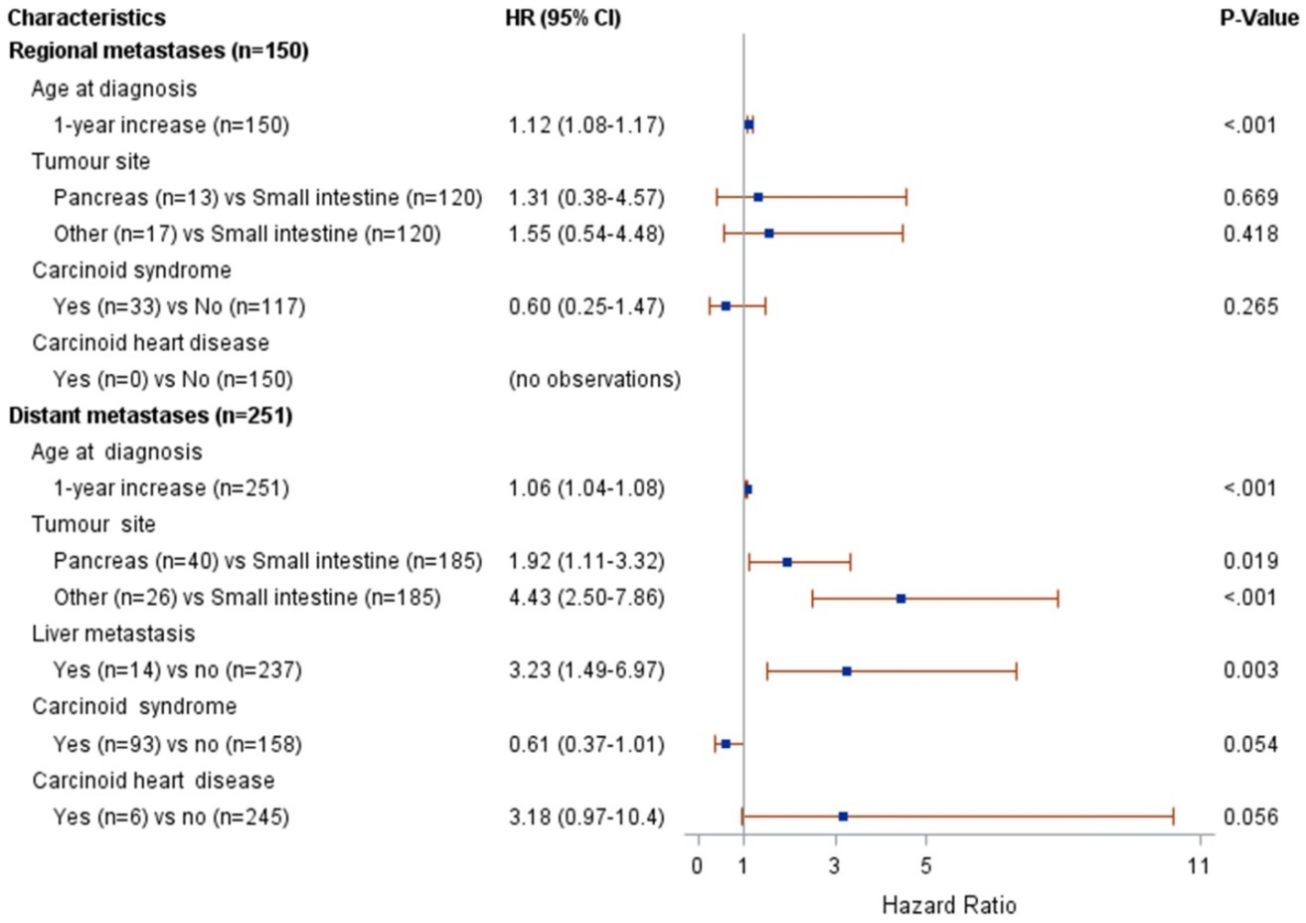
Results from multivariate Cox modelling of clinical characteristics and risk of death.

Figure 2 presents the results from the univariate Cox modelling. The following clinical characteristics at diagnosis were associated with a higher risk of death (p<0.05): pancreatic or other NET (compared to small intestinal NET), distant metastases (compared to regional metastases or unknown stage) and the absence of carcinoid syndrome diagnosis. The following potential confounders were associated with risk of death (p<0.05): age at diagnosis, lymph node metastasis, liver metastasis and bone metastasis. The interaction between stage and age at diagnosis was associated with risk of death at p=0.004; therefore, the multivariate modelling was performed by stage (regional vs. distant metastases). The results from the multivariate Cox modelling are shown in Figure 3. For patients with regional metastases, a 1-year increase in age at diagnosis was associated with a higher risk of death (HR 1.12; 95% CI 1.08-1.17); no other variable was significantly associated with risk of death. For patients with distant metastases, a 1-year increase in age at diagnosis was associated with a higher risk of death (HR 1.06; 95% CI 1.04-1.08). Compared to small intestinal NET, pancreatic NET was associated with a higher risk of death (HR 1.9; 95% CI 1.1-3.3), as was other NET (HR 4.4; 95% CI 2.5-7.9). Presence of liver metastases also increased the risk of death (HR 3.2; 95% CI 1.5-7.0). The limited number of patients fulfilling some of these clinical characteristics should be noted (patient numbers are presented in Figure 3).

#### Risk of death in relation to treatment status

The following subgroups were compared in the analyses of patients with first-line treatment only: surgery of the primary tumour site (n=142) and SSA (n=50). The median survival time among patients who had surgery of the primary tumour site was 6.2 (95% CI 5.7-not reached) years. For SSA, the corresponding figure was 4.1 (95% CI 1.7-6.1) years. In the univariate survival analysis, patients with first-line SSA had an increased risk of death compared to those with surgery of the primary site (HR 2.2; 95% CI 1.3-3.8, p=0.004). When adjusting for potential confounders (age at diagnosis, liver metastases, year of diagnosis and stage), this association was no longer statistically significant.

**Figure 4:**
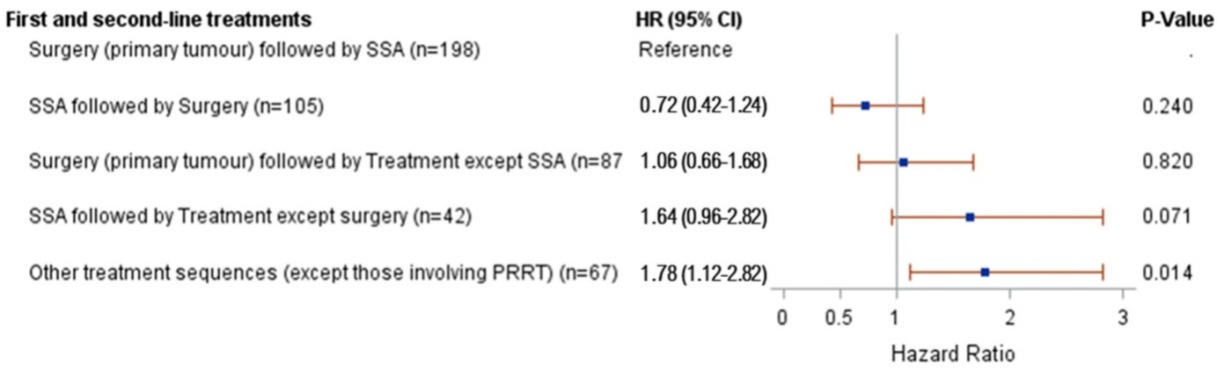
Results from univariate Cox modelling of first- and second-line treatments and risk of death.

The results from the univariate survival analysis of first- and second-line treatments and survival are presented in Figure 4. The risk of death was higher for patients with “other treatment sequences” than for patients with surgery of the primary tumour site followed by SSA (HR 1.8; 95% CI 1.1-2.8). The risk of death was not statistically different for any of the remaining treatment sequences compared to patients with surgery of the primary tumour site followed by SSA. The one treatment sequence for which a lower risk of death was suggested was SSA followed by surgery (compared to surgery followed by SSA), although this association was not statistically significant (HR 0.72; 95% CI 0.4-1.2). However, a higher percentage of patients with surgery as first-line treatment had small intestinal NET (61%), compared to 27% among patients with SSA as first-line. When adjusting for potential confounders, there were no statistically significant associations between treatment sequence and risk of death.

## Discussion

This population-based study presents real-world evidence on treatment patterns, including treatment sequences, and the overall survival among patients diagnosed with metastatic GEP-NET (grade 1 or 2) in Sweden.

The clinical characteristics of the patients included in this study are in line with previous findings concerning patients with metastatic or advanced GEP-NET 2, 27, 28, 30, 31. At diagnosis, carcinoid syndrome had been diagnosed among 36% of patients in the National population. In the Regional population, a subpopulation to the National population, 74% of patients had been diagnosed with carcinoid syndrome at time of GEP-NET diagnosis. The difference is suggestive of regional variations in the coding practices applied for the diagnosis of carcinoid syndrome.

According to Nordic and European guidelines for the treatment of GEP-NET, all patients with GEP-NET should be considered for surgery, when possible 5, 32. In the current study, most patients had undergone surgery for metastatic GEP-NET. Furthermore, surgery was the most common first-line treatment, and this was performed in close connection to the diagnosis. Surgery of the primary tumour site was more common in small intestinal NET than in pancreatic NET, in agreement with previous findings 8.

First-line surgery was typically followed by SSA or no further treatment. However, it is possible that perioperative use of SSA may not have been captured to a full extent, if this was administered by health care personnel (and not purchased by the patient via a prescription). In the Regional population, patients with functioning tumours more often received SSA after first-line surgery than patients with non-functioning tumours, possibly explained by the fact that during most of the study period, SSA was only indicated for symptom control and not as an antitumoural treatment.

The next most common first-line treatment was SSA, and the majority of these patients initiated their SSA less than 2 months after diagnosis (median). Most patients with first-line SSA had surgery as second-line treatment.

Clinical experience shows that HAE is more commonly used at the Sahlgrenska University Hospital (thus, among the patients included in the Regional population), as compared to the rest of Sweden. The results for the Regional population were thus in line with our expectations.

Even though the possibility for direct comparison is made difficult by the fact that the Regional population is a subgroup of the National population, the study suggests that there are regional differences in therapeutic traditions and treatment patterns in Sweden. Very likely there will also be regional variations in coding practices of medical and surgical interventions, although all procedure codes registered in the data were reviewed. Furthermore, the eligibility criteria were further assessed in the Regional population using the in-depth data available via the medical chart review. This led to the exclusion of 14 patients not fulfilling the criteria (i.e. 10% of the 141 originally included patients). Seven of these 14 patients were excluded due to having a grade 3 tumour, as defined by Ki 67 index >20%. This suggest that about 5% of the patients in the National population may have had a grade 3 tumour, but were misclassified as having grade 1 or 2 disease. Since these in-depth data (e.g. Ki 67 index) were not available on a national level, the corresponding patients were not possible to exclude from the National population, and this is a limitation of the study.

The median survival time was 7.0 years from the date of metastatic GEP-NET diagnosis, and the 5-year survival rate was 63%. This is in agreement with previous findings 3. Survival tended to be longer for small intestinal NET as compared to both pancreatic NET and other GEP-NET; this has also been reported previously 3. For patients treated with first-line only, survival tended to be longer for patients who had undergone surgery as compared to SSA. A longer survival among surgically treated patients has also been reported previously, although not specifically reporting on its place in the treatment sequence 7. Patients treated with SSA followed by surgery tended to have longer survival than those treated with surgery followed by SSA. However, the patient populations were not entirely comparable (e.g. for patients with surgery as first-line, 61% had small intestinal NET as compared to 27% of patients with SSA as first-line), which might bias the interpretation.

Based on multivariate modelling, pancreatic NET was associated with an increased risk of death compared to small intestinal NET for patients with distant metastases (HR 1.9; 95% CI 1.1-3.3) but not for patients with only regional metastases. These findings are in agreement with previous findings concerning patients with GEP-NET (irrespective of metastatic status) 3. In the current study, having liver metastases increased the risk of death compared to other distant metastases (HR 3.2; 95% CI 1.5-7.0). Previous studies have also shown that having distant metastases is a predictor of poorer survival 26-28, 33. However, the findings in the current study has to be interpreted with some caution as the low occurrence of liver metastases among patients with distant metastases may indicate underreporting of diagnoses related to metastases in the National Patient Register. The lack of conclusion for regional metastases (as compared to distant metastases) could also be linked to insufficient power for the analysis performed.

The observed protective effect of carcinoid syndrome on survival in the univariate analyses could indicate unspecific use of this diagnosis code. Another possibility would be earlier diagnosis and treatment of the disease due to presence of symptomatic disease in the form of carcinoid syndrome.

### Strengths and limitations

This study was based on real-world data from national registers. All Swedish citizens are covered in the registers and the data are based on clinical practice. This enabled the inclusion of a large patient population identified from multiple clinical sites. Furthermore, the registers have been available for a long period of time, which enabled longitudinal analyses of treatment patterns and survival. Due to the unique personal identification numbers, the linkage between registers was performed with high precision. All diagnostic codes were validated by the clinical experts. The study was also complemented with disease-specific data from medical charts in a subsample of the study population, contributing with more in-depth data.

There are also some limitations. Since the data are based on clinical practice, the quality of the diagnostic coding may vary between hospitals and/or physicians. However, all diagnostic codes were validated as presented above. As there are no nationally agreed coding algorithms for morphological type in the data for the Cancer Register, the coding may have varied between the regional cancer centres. The extended review of eligibility criteria performed during the medical chart review revealed that some of the included patients (10%) did not fulfil the criteria. While these patients were excluded from the analyses based on the medical chart review in the Regional population, this was not possible to correct based on the data available only in the registers for the National population. This suggests that the National population may include some patients who did not fulfil the eligibility criteria. Further, it implies, with regards to the completeness and validity of data for GEP-NET in the Cancer Register, that there is room for improving the quality of reporting for this cancer form. The Swedish Cancer Register is generally considered to be of good quality, but quality of reporting may vary for different tumour sites, patient age, and could change over time. To the authors' knowledge, there are no studies on the validity and completeness specific for GEP-NET in the Cancer Register, but other cancer forms have been evaluated. A study estimating the overall coverage of malignant cancer cases in the Cancer Register in 1998 found that the overall completeness of the register was high and comparable with other high-quality registers in Northern Europe 34. However, the degree of underreporting was tumour site specific, increased with age, and may not be random, as diagnoses without histology or cytology verification were overrepresented 34. Other publications have also described under-reporting of cases to the Cancer Register for acute leukemias and central nervous system tumours, and that reporting varied with tumour site 35, 36. Results obtained by comparing data in the Cancer Register and the Swedish Register on Palliative Care indicated that approximately 12% of patients dying of cancer in palliative care are not reported to the Cancer Register, that specialized hospital departments diagnosed the majority of the unreported patients, and that routines for reporting patients to the Cancer Register based on radiological findings should be revised 37. In patients with surgically treated oesophageal cancer, the validity for tumour stage was high as reported in the Cancer Register (determined by comparing data in the Cancer Register with comprehensive tumour stage data based on pathological TNM). However, the recording of pathological TNM stage and the individual components of TNM could be improved for oesophageal cancer in the register 38.

Furthermore, it is possible that the exclusion of patients with likely grade 3 tumours may have led to the exclusion of some patients with grade 1 or 2. The relatively low occurrence of metastases observed in the data also suggest that some patients who actually had metastases at diagnosis had not received such a diagnosis in the National Patient Register or the Cancer Register and were therefore not captured. Therefore, it is likely that the population included in this study is smaller than the total target population of patients with metastatic GEP-NET of grade 1 or 2. In addition, the sample size was limited for some of the analyses (e.g. PRRT).

There is a possibility that patients with a diagnosis of metastatic GEP-NET grade 1 or 2 after 1 July 2005 also have another NET diagnosis, or other malignancy, prior to or after the date of original diagnosis. The Cancer Register is a mandatory register, and all tumours considered to be a new primary tumour should be reported, meaning that the same patient might be included in the register more than once. Inclusions of patients with NET diagnosis prior to 2005 could have an impact on the survival analyses, but the extent of this is unknown.

## Conclusions

The most common first-line treatment in Swedish patients with metastatic GEP-NET (grade 1 or 2) was surgery, performed in close connection with the diagnosis. First-line surgery was typically followed by SSA or no further treatment. Among patients with distant metastases, pancreatic NET (vs. small intestinal NET) and liver metastases were associated with a poorer survival.

## Figures and Tables

**Table 1:** Clinical characteristics at diagnosis.

	National population^1)^(n=811)	Regional population^1)^(n=127)
**Age at diagnosis (years), mean (SD)**	66.2 (12.2)	65.6 (11.7)
**Sex, n (%)**		
Male	423 (52.2%)	64 (50.4%)
Female	388 (47.8%)	63 (49.6%)
**Tumour site, n (%)**		
Small intestine	615 (75.8%)	102 (80.3%)
Pancreas	85 (10.5%)	18 (14.2%)
Other (stomach, colon, rectum)	111 (13.7%)	7 (5.5%)
**Metastatic sites^2)^** (most common)**, n (%)**		
Liver	183 (59.6%)	41 (78.8%)
Lymph nodes	131 (42.7%)	21 (40.4%)
Intestine and peritoneum	34 (11.1%)	n<5
Missing data, n	504	75
**Stage^3)^, n (%)**		
Localized disease	15 (3.6%)	n<5
Regional metastases	150 (36.1%)	n<5
Distant metastases	251 (60.3%)	46 (90.2%)
Unknown or missing data, n	395	76
**Carcinoid syndrome^4)^, n (%)**	293 (36.1%)	94 (74.0%)
**Carcinoid heart disease^5)^, n (%)**	10 (1.2%)	n<5
**Time from GEP-NET diagnosis until death or 31 Dec 2013 (years), mean (SD)**	3.18 (2.22)	2.97 (1.87)
***Data from chart review***		
**Functioning status^6)^** (missing: n=4)	n/a	
No symptoms		61 (49.6%)
Symptoms		62 (50.4%)
**Liver burden^7)^** (missing: n=2)	n/a	
None		37 (29.6%)
Low		79 (63.2%)
High		9 (7.2%)
**Ki 67 index** (missing: n=2)	n/a	
Ki 67 index <3%		90 (72.0%)
Ki 67 index 3-20%		35 (28.0%)
**5-HIAA level^8)^** (missing: n=17)	n/a	
Below ULN		33 (30.0%)
Above ULN		77 (70.0%)
**CgA level** (missing: n=2)	n/a	
Below ULN		11 (8.8%)
Above ULN		114 (91.2%)

5-HIAA: 5-hydroxyindoleacetic acid; CgA: chromogranin A; GEP-NET: gastroenteropancreatic neuroendocrine tumours; ICD-10: International Classification of Diseases 10^th^ revision; NET: neuroendocrine tumours; SD: standard deviation; ULN: upper limit of normal. Footnotes: 1) The Regional population is a subgroup of the National population; hence, the National population and the Regional population should not be compared. 2) Data on metastatic site was available for 307 patients in the National population and for 52 patients in the Regional population. Percentages were based on the number of non-missing observations. A patient could have more than one metastatic site registered. 3) Based on TNM-codes in the Cancer Register; Localized N=0 and M=0, Regional N=1-3 unless M=1, Distant M=1. Data on stage was available for 416 patients in the National population and for 51 patients in the Regional population. Percentages were based on the number of non-missing observations. 4) ICD-10 code E34.0 within 6 months from GEP-NET diagnosis. 5) ICD-10 codes I36 or I39.2 within 6 months from GEP-NET diagnosis. 6) Categorised based on presence of hormonal symptoms or not, according to clinical judgement (i.e. classified as a functioning tumour if the patient experienced hormonal symptoms). 7) Categorised as high if the tumour mass corresponded to >50% of the overall liver size or if the metastasis was described as "massive" or alike. 8) 24-hour value.

**Table 2:** Occurrence of treatments at any time during the observation period.

	National population^1)^(n=811)		Regional population^1)^(n=127)	
	n (%)	95% CI	n (%)	95% CI
**Any treatment**	714 (88.0%)	85.6-90.2	126 (99.2%)	95.7-100.0
				
**Surgery**				
Any surgery	584 (72.0%)	68.8-75.1	112 (88.2%)	81.3-93.2
Primary site	542 (66.8%)	63.5-70.1	105 (82.7%)	75.0-88.8
Gall or bile ducts	143 (17.6%)	15.1-20.4	58 (45.7%)	36.8-54.7
Lymph nodes	97 (12.0%)	9.8-14.4	47 (37.0%)	28.6-46.0
Liver	81 (10.0%)	8.0-12.3	11 (8.7%)	4.4-15.0
Peritoneum	22 (2.7%)	1.7-4.1	12 (9.4%)	5.0-15.9
Other	10 (1.2%)	0.6-2.3	n<5	n<5
				
**Locoregional interventions**				
HAE	80 (9.9%)	7.9-12.1	40 (31.5%)	23.5-40.3
RFA	75 (9.2%)	7.3-11.5	9 (7.1%)	3.3-13.0
				
**PRRT**	66 (8.1%)	6.3-10.2	11 (8.7%)	4.4-15.0
				
**Pharmacological treatment**				
SSA	470 (58.0%)	54.5-61.4	95 (74.8%)	66.3-82.1
IFN-alpha	148 (18.2%)	15.6-21.1	22 (17.3%)	11.2-25.0
Chemotherapy	93 (11.5%)	9.4-13.9	13 (10.2%)	5.6-16.9
Molecular targeted therapy	24 (3.0%)	1.9-4.4	6 (4.7%)	1.8-10.0

HAE: hepatic artery embolization; IFN: interferon; PRRT: peptide receptor radionuclide therapy; RFA: radiofrequency ablation; SSA: somatostatin analogues. Footnote: 1) The Regional population is a subgroup of the National population; hence, the National population and the Regional population should not be compared.

**Table 3:** First-line treatments.

	National population^1)^(n=811)	Regional population^1)^(n=127)
**Surgery**		
n (%)	460 (56.7%)	83 (65.4%)
(95% CI for %)	(53.2-60.2)	(56.4-73.6)
Time to treatment (months), mean (median)	0.8 (0.0)	0.9 (0.0)
(95% CI for mean)	(0.6-1.0)	(0.4-1.4)
**SSA^2)^**		
n (%)	201 (24.8%)	36 (28.3%)
(95% CI for %)	(21.8-27.9)	(20.7-37.0)
Time to treatment (months), mean (median)	3.0 (1.7)	2.1 (1.6)
(95% CI for mean)	(2.2-3.8)	(1.5-2.7)
**IFN-alpha**		
n (%)	10 (1.2%)	n<5
(95% CI for %)	(0.6-2.3)	
Time to treatment (months), mean (median)	2.1 (2.4)	n<5
(95% CI for mean)	(1.3-2.9)	
**Chemotherapy**		
n (%)	31 (3.8%)	n<5
(95% CI for %)	(2.6-5.4)	
Time to treatment (months), mean (median)	4.3 (2.3)	n<5
(95% CI for mean)	(2.0-6.5)	

CI: confidence interval; IFN: interferon; SSA: somatostatin analogues. Footnotes: 1) The Regional population is a subgroup of the National population; hence, the National population and the Regional population should not be compared. 2) Perioperative use of SSA may not have been captured to a full extent, if this was administered by health care personnel (and not purchased by the patient via a prescription).

**Table 4:** Second-line treatments.

	National population^1)^(n=811)		Regional population^1)^(n=127)	
	**n (%)**	**Time to second-line (months)Mean (median); (95% CI for mean)**	**n (%)**	**Time to second-line (months)Mean (median); (95% CI for mean)**
**First-line: Surgery**	**N=460**		**N=83**	
Second-line treatment				
SSA^2)^	212 (46.1%)	5.7 (1.3)(4.1-7.3)	51 (61.4%)	1.6 (0.5)(0.6-2.6)
IFN-alpha	28 (6.1%)	3.4 (3.2)(2.8-4.0)	n<5	n<5
Chemotherapy	19 (4.1%)	3.3 (2.3)(1.8-4.8)	n<5	n<5
Locoregional	8 (1.7%)	4.9 (3.9)(2.5-7.4)	6 (7.2%)	5.8 (5.7)(2.8-8.8)
Other	7 (1.5%)	n/a	n<5	n/a
No second-line treatment	186 (40.0%)	n/a	22 (26.5%)	n/a
**First-line: SSA^2)^**	**N=201**		**N=36**	
Second-line treatment				
Surgery	105 (52.2%)	2.1 (1.5)(1.6-2.6)	27 (75.0%)	1.5 (1.1)(1.0-1.9)
IFN-alpha	23 (11.4%)	5.4 (1.8)(1.6-9.3)	n<5	n<5
Chemotherapy	11 (5.5%)	5.4 (2.5)(0.0-12.0)	n<5	n<5
Other	8 (4.0%)	n/a	n<5	n/a
No second-line treatment	54 (26.9%)	n/a	6 (16.7%)	n/a

CI: confidence interval; IFN: interferon; SSA: somatostatin analogues. Footnotes: 1) The Regional population is a subgroup of the National population; hence, the National population and the Regional population should not be compared. 2) Perioperative use of SSA may not have been captured to a full extent, if this was administered by health care personnel (and not purchased by the patient via a prescription).

## Abbreviations

5-HIAA: 5-Hydroxyindoleacetic acid
CgA: chromogranin A
CI: confidence intervals
GEP-NET: gastroenteropancreatic neuroendocrine tumour
GI: gastrointestinal
HAE: hepatic artery embolization
HR: hazard ratio
ICD-O/3: International Classification of Diseases for Oncology, 3^rd^ edition
IFN-alpha: interferon alpha
MWA: microwave ablation
OS: overall survival
PPI: proton-pump inhibitors
PRRT: peptide receptor radionuclide therapy
RFA: radiofrequency ablation
SIRT: selective internal radiation therapy
SSA: somatostatin analogues
TNM: Tumour, Node, Metastases
ULN: upper limit of normal

## References

[1] Warner RR (2005). Enteroendocrine tumors other than carcinoid: a review of clinically significant advances. Gastroenterology.

[2] van Cutsem E. Neuroendocrine tumors - Diagnostic and therapeutic challenges: introduction.

[3] Lawrence B, Gustafsson BI, Chan A, Svejda B, Kidd M, Modlin IM (2011). The epidemiology of gastroenteropancreatic neuroendocrine tumors. Endocrinol Metab Clin North Am.

[4] Massironi S, Sciola V, Peracchi M, Ciafardini C, Spampatti MP, Conte D (2008). Neuroendocrine tumors of the gastro-entero-pancreatic system. World J Gastroenterol.

[5] Oberg K, Knigge U, Kwekkeboom D, Perren A (2012). Neuroendocrine gastro-entero-pancreatic tumors: ESMO Clinical Practice Guidelines for diagnosis, treatment and follow-up. Ann Oncol.

[6] Cidon EU (2017). New therapeutic approaches to metastatic gastroenteropancreatic neuroendocrine tumors: A glimpse into the future. World J Gastrointest Oncol.

[7] O'Connor JM, Marmissolle F, Bestani C, Pesce V, Belli S, Dominichini E (2014). Observational study of patients with gastroenteropancreatic and bronchial neuroendocrine tumors in Argentina: Results from the large database of a multidisciplinary group clinical multicenter study. Mol Clin Oncol.

[8] Niederle MB, Niederle B (2011). Diagnosis and treatment of gastroenteropancreatic neuroendocrine tumors: current data on a prospectively collected, retrospectively analyzed clinical multicenter investigation. Oncologist.

[9] Garcia-Carbonero R, Capdevila J, Crespo-Herrero G, Diaz-Perez JA, Martinez Del Prado MP, Alonso Orduna V (2010). Incidence, patterns of care and prognostic factors for outcome of gastroenteropancreatic neuroendocrine tumors (GEP-NETs): results from the National Cancer Registry of Spain (RGETNE). Ann Oncol.

[10] Thomas D, Tsolakis AV, Grozinsky-Glasberg S, Fraenkel M, Alexandraki K, Sougioultzis S (2013). Long-term follow-up of a large series of patients with type 1 gastric carcinoid tumors: data from a multicenter study. Eur J Endocrinol.

[11] Kulke MH, Benson AB, Dasari A, Huynh L, Cai B, Totev T (2019). Real-World Treatment Patterns and Clinical Outcomes in Advanced Gastrointestinal Neuroendocrine Tumors (GI NET): A Multicenter Retrospective Chart Review Study. Oncologist.

[12] Alese OB, Jiang R, Shaib W, Wu C, Akce M, Behera M (2019). High-Grade Gastrointestinal Neuroendocrine Carcinoma Management and Outcomes: A National Cancer Database Study. Oncologist.

[13] Jiao X, Pulgar S, Boyd M, Braiteh F, Mirakhur B, Pitman Lowenthal S (2018). Treatment Patterns and Clinical Outcomes in Patients With Metastatic Gastroenteropancreatic Neuroendocrine Tumors Treated in the Community Practice Setting in the United States. Pancreas.

[14] Benson AB, III, Broder MS, Cai B, Chang E, Neary MP, Papoyan E (2017). Real-world treatment patterns of gastrointestinal neuroendocrine tumors: A claims database analysis. World J Gastroenterol.

[15] Herring M, Huynh L, Duh MS, Vekeman F, Tiew A, Neary M (2017). Real-world treatment patterns in advanced pancreatic neuroendocrine tumors in the era of targeted therapy: perspectives from an academic tertiary center and community oncology practices. Med Oncol.

[16] Chuang CC, Bhurke S, Chen SY, Brulais S, Gabriel S (2015). Clinical characteristics, treatment patterns, and economic burden in patients treated for neuroendocrine tumors in the United States: a retrospective cohort study. J Med Econ.

[17] Strosberg J, Casciano R, Stern L, Parikh R, Chulikavit M, Willet J (2013). United States-based practice patterns and resource utilization in advanced neuroendocrine tumor treatment. World J Gastroenterol.

[18] Kim SJ, Kim JW, Han SW, Oh DY, Lee SH, Kim DW (2010). Biological characteristics and treatment outcomes of metastatic or recurrent neuroendocrine tumors: tumor grade and metastatic site are important for treatment strategy. BMC Cancer.

[19] Lokesh KN, Anand A, Lakshmaiah KC, Babu KG, Lokanatha D, Jacob LA (2018). Clinical profile and treatment outcomes of metastatic neuroendocrine carcinoma: A single institution experience. South Asian J Cancer.

[20] Hafeez U, Joshi A, Bhatt M, Kelly J, Sabesan S, Vangaveti V (2017). Clinical profile and treatment outcomes of advanced neuroendocrine tumours in rural and regional patients: a retrospective study from a regional cancer centre in North Queensland, Australia. Intern Med J.

[21] Fisher MD, Pulgar S, Kulke MH, Mirakhur B, Miller PJ, Walker MS (2018). Treatment Outcomes in Patients with Metastatic Neuroendocrine Tumors: a Retrospective Analysis of a Community Oncology Database. J Gastrointest Cancer.

[22] McMullen T, Al-Jahdali A, de Gara C, Ghosh S, McEwan A, Schiller D (2017). A population-based study of outcomes in patients with gastrointestinal neuroendocrine tumours. Can J Surg.

[23] Aytac E, Ozdemir Y, Ozuner G (2014). Long term outcomes of neuroendocrine carcinomas (high-grade neuroendocrine tumors) of the colon, rectum, and anal canal. J Visc Surg.

[24] Pape UF, Berndt U, Muller-Nordhorn J, Bohmig M, Roll S, Koch M (2008). Prognostic factors of long-term outcome in gastroenteropancreatic neuroendocrine tumours. Endocr Relat Cancer.

[25] Arnold R, Wilke A, Rinke A, Mayer C, Kann PH, Klose KJ (2008). Plasma chromogranin A as marker for survival in patients with metastatic endocrine gastroenteropancreatic tumors. Clin Gastroenterol Hepatol.

[26] Bergestuen DS, Aabakken L, Holm K, Vatn M, Thiis-Evensen E (2009). Small intestinal neuroendocrine tumors: prognostic factors and survival. Scand J Gastroenterol.

[27] Yao JC, Hassan M, Phan A, Dagohoy C, Leary C, Mares JE (2008). One hundred years after "carcinoid": epidemiology of and prognostic factors for neuroendocrine tumors in 35,825 cases in the United States. J Clin Oncol.

[28] Ter-Minassian M, Chan JA, Hooshmand SM, Brais LK, Daskalova A, Heafield R (2013). Clinical presentation, recurrence, and survival in patients with neuroendocrine tumors: results from a prospective institutional database. Endocr Relat Cancer.

[29] Verbeek WH, Korse CM, Tesselaar ME (2016). GEP-NETs UPDATE: Secreting gastro-enteropancreatic neuroendocrine tumours and biomarkers. Eur J Endocrinol.

[30] Ahmed A, Turner G, King B, Jones L, Culliford D, McCance D (2009). Midgut neuroendocrine tumours with liver metastases: results of the UKINETS study. Endocr Relat Cancer.

[31] Larsson G SP, Oberg K, Eriksson B, von Essen L (2001). Health-related quality of life, anxiety and depression in patients with midgut carcinoid tumours. Acta Oncol.

[32] Janson ET, Sorbye H, Welin S, Federspiel B, Gronbaek H, Hellman P (2010). Nordic Guidelines 2010 for diagnosis and treatment of gastroenteropancreatic neuroendocrine tumours. Acta Oncol.

[33] Janson ET, Holmberg L, Stridsberg M, Eriksson B, Theodorsson E, Wilander E (1997). Carcinoid tumors: analysis of prognostic factors and survival in 301 patients from a referral center. Ann Oncol.

[34] Barlow L, Westergren K, Holmberg L, Talback M (2009). The completeness of the Swedish Cancer Register: a sample survey for year 1998. Acta Oncol.

[35] Astrom M, Bodin L, Tidefelt U (2001). Adjustment of incidence rates after an estimate of completeness and accuracy in registration of acute leukemias in a Swedish population. Leuk Lymphoma.

[36] Tettamanti G, Ljung R, Ahlbom A, Talback M, Lannering B, Mathiesen T (2019). Central nervous system tumor registration in the Swedish Cancer Register and Inpatient Register between 1990 and 2014. Clin Epidemiol.

[37] Nilsson M, Tavelin B, Axelsson B (2014). A study of patients not registered in the Swedish Cancer Register but reported to the Swedish Register of Palliative Care 2009 as deceased due to cancer. Acta Oncol.

[38] Brusselaers N, Vall A, Mattsson F, Lagergren J (2015). Tumour staging of oesophageal cancer in the Swedish Cancer Registry: A nationwide validation study. Acta Oncol.

